# Supplementary material for: Associations of breastfeeding history with metabolic syndrome and cardiovascular risk factors in community-dwelling parous women: The Japan Multi-Institutional Collaborative Cohort Study
Source: PLoS One. 2022 Jan 19;17(1):e0262252. doi: 10.1371/journal.pone.0262252 (PMC8769371; doi:10.1371/journal.pone.0262252)
Supplement: Attachment — Submitted filename: Response To Reviewers.docx [file pone.0262252.s004.docx]

June 5, 2021

Antonio Palazón-Bru, Ph.D.

Academic Editor

*PLOS ONE*

Dear Dr. Palazón-Bru,

Thank you for inviting us to submit our revised manuscript entitled, “Associations of breastfeeding history with metabolic syndrome and cardiovascular risk factors in community-dwelling parous women: The Japan Multi-Institutional Collaborative Cohort Study” to *PLOS ONE* (Reference Number: PONE-D-21-02465). We also appreciate the time and effort you and the reviewers have dedicated to providing feedback. We have incorporated modifications that reflect the suggestions you have provided. We hope that our edits and the responses we provide below satisfactorily address all the issues and concerns you and the reviewers have noted.

To facilitate your review of our revisions, the following are point-by-point responses to the questions and comments delivered in your letter dated February 23, 2021. Amendments that we have made are shown in colored text throughout the Revised Article with Track Changes.

Academic Editor’s comments

(1) *Please ensure that your manuscript meets PLOS ONE's style requirements, including those for file naming.*

**Response:** We have embedded the editable tables and inserted the captions of the tables and a figure in the text of the manuscript. We have also named our files according to the journal’s requirements.

(2) *Please refer to the specific statistical analyses performed as well as any post-hoc corrections to correct for multiple comparisons. If these were not performwed please justify the reasons.…Additionally, please ensure you have thoroughly discussed any potential limitations of this study within the Discussion.*

**Response:** In the revised manuscript, we have mentioned that our primary outcome was metabolic syndrome and secondary outcomes were cardiovascular risk factors (lines 65–67 and 168–169 in the unmarked manuscript). We conducted Bonferroni corrections for multiple comparisons in the trend tests examining the association between breastfeeding history and metabolic syndrome, because we used three indices of breastfeeding history (the longest breastfeeding duration, the number of breastfed children, and the total breastfeeding duration). Regarding the tests examining the association between categorized breastfeeding history and metabolic syndrome, we have not conducted any corrections for multiple comparisons because observational epidemiological studies rarely conduct these corrections on such analyses (lines 266–276). In the Discussion, we have added potential limitations of the present study including multiple comparisons as well as potential measurement errors and misclassification in breastfeeding history, reverse causality, and recall bias (lines 464–466 and 493–513).

(3) *We note that you have included the phrase “data not shown” in your manuscript. Unfortunately, this does not meet our data sharing requirements. PLOS does not permit references to inaccessible data. We require that authors provide all relevant data within the paper, Supporting Information files, or in an acceptable, public repository.*

**Response:** We have added a supplementary table (S1 Table) reporting the associations between body mass index at about 20 years old and breastfeeding history and have mentioned the methods of statistical analysis (lines 243–265) and results (lines 399–402) in the revised manuscript.

(4) *We note that you have indicated that data from this study are available upon request. PLOS only allows data to be available upon request if there are legal or ethical restrictions on sharing data publicly.*

**Response:** The data used in the present study cannot be made publicly available because the participants of the present study have not given informed consent for public data sharing and the ethics committee of the Nagoya University Graduate School of Medicine does not approve the sharing. To obtain access to the confidential data, interested researchers can contact the Administration Office of the ethics committee of the Nagoya University Graduate School of Medicine (iga-shinsa@adm.nagoya-u.ac.jp). After receiving a request, informed consent from the participants will be sought.

Reviewer #1’s comment

(1) *the main concern is that the conclusion does not seem justified and supported by the results. Specifically, the reported associations do not seem to be significant.*

**Response:** Our results consistently indicated that breastfeeding history may be inversely related to the prevalence of metabolic syndrome and cardiovascular risk factors, especially in participants <55 years old. Even after Bonferroni corrections, the number of breastfed children was significantly associated with lower prevalence of metabolic syndrome in participants <55 years old.

Reviewer #2’s comments

(1) *Lines 95-98 Is there more recent data that can be referenced than reference 7?*

**Response:** More recent analyses using the data from the Global Burden of Disease have not reported the combined effect of high blood pressure, blood glucose, serum cholesterol, and body mass index on death from cardiovascular disease, chronic kidney disease, and diabetes (Lancet. 2017;390(10100):1345-1422. and Lancet. 2018;392(10159):1923-1994.) Thus, we have retained the article cited in the previous manuscript (lines 92–95).

(2) *Line 235 Was presenting breastfeeding in terms of months considered rather than weeks? Weeks may be less intuitive to relate to in terms of breastfeeding duration, as breastfeeding recommendations are usually expressed in months.*

**Response:** We have changed our breastfeeding duration measured in weeks to that measured in months throughout the manuscript including the tables.

(3) *Generally, the discussion was very descriptive in various instances but could benefit from being more concise overall.*

**Response:** In the revised manuscript, we have reduced the descriptions about cardiovascular risk factors because they were the secondary outcomes in the present study, and our analysis provided generally consistent results with previous studies about these risk factors (lines 378–381 and lines 454–466).

(4) *Line 318 I would suggest being more careful with interpretation and language used (e.g. use "may have" rather than "probably have"). There could be other factors besides age to be considered and discussed, and that can explain some of the differences observed such as sample size, sample characteristics, follow-up periods, etc.*
(5) *Lines 319-324 The findings from the study need to be discussed in light of the evidence provided by other studies and a comment on the study design of these studies should be included. How much of the evidence was from longitudinal/cross-sectional studies?*

**Response:** According to the reviewers’ recommendation, we have corrected the language about the influence of age (lines 414–415). Furthermore, we have explained the differences in sample size, the proportions of participants with breastfeeding history and metabolic syndrome, and study design between our study and previous studies (lines 427–440).

(6) *Lines 324-326 Again, did this evidence come from mainly longitudinal or cross-sectional studies? Has there been any systematic review examining whether age affects associations of breastfeeding with cardiovascular risk factors or cardiovascular disease?*

**Response:** In the revised manuscript, we have mentioned which associations come from case-control or cohort studies and that no systematic review or meta-analysis has been conducted about the effect modification by age on the associations of breastfeeding history with cardiovascular risk factors or cardiovascular disease (lines 418–425).

(7) *What about the limitation of the breastfeeding variable which was a product of the number of breastfed children and longest breastfeeding duration? Breastfeeding duration may have varied among children from the same mother, and the breastfeeding duration used in the study may not accurately reflect the true overall breastfeeding duration across children. Please add this limitation/discuss.*

**Response:** We have added the potential limitation of how we calculated total breastfeeding duration in the Discussion of the revised manuscript (lines 498–501).

(8) *Acknowledging the limitations of cross-sectional evidence, what future studies are needed for further inform the evidence base and extend these findings? Please add this to the discussion.*
(9) *The conclusions should remind readers that this study provides evidence of a cross-sectional nature, which limits inferences and statements that are too conclusive. Please reword accordingly.*

**Response:** In the Discussion of the revised manuscript, we have added what studies should be conducted in the future to clarify the associations between breastfeeding history and metabolic syndrome (lines 511–513), and have modified the conclusion (lines 514–520).

(10) *Please add that this is a cross-sectional study in the Methods of the Abstract.*

**Response:** We have stated that our study is a cross-sectional design in the Methods of the Abstract in the revised manuscript (line 60).

Reviewer #3’s comments

(1) *The abstract needs review. The purpose of the abstract should be the same as that described at the end of the introduction. In my opinion, the abstract is more correct according to the study. In the methods, it is also important to highlight the breastfeeding categories already in this part of the manuscript. The abstract is long, but the results incomplete. The authors mention the sample starting at 10,432, which creates confusion. Still in the results I suggest that the way of presenting the data with measure of effect (OR) and their respective 95% CI should be as follows, for example: [OR = 0.57 (95% CI 0.36; 0.89)]. This comment applies to the abstract and all text. The measure of effect used in the study, obtained through logistic regression, was an odds ratio (ratio). Therefore, the interpretation of the results mentioning lower prevalence is totally wrong. In this case it would be "lower odds". (see line 78 of the abstract).*
(2) *Review carefully so that the abstract objective and introduction are compatible.*

**Response:** We have made the following revisions. First, we have modified the Objective of the Abstract to include examining the effect modification by age to align the aim with that in the Introduction (lines 56–58). Second, we have explained the categories of breastfeeding duration in the Methods section of the Abstract (lines 64–65). Third, we have again indicated that our total sample size was 11,118 women in the Results section of the Abstract (lines 71–72). Fourth, we have revised the descriptions about the results of logistic regression analysis throughout the revised manuscript to correct the interpretation of the results.

(3) *It would be interesting for the authors to evaluate the time of exclusive breastfeeding separately from the time of total breastfeeding. There is evidence that shows the influence of EBF on cardiovascular outcomes, for example. I suggest the following reference: https://www.nature.com/articles/s41366-018-0317-5.*

**Response:** Unfortunately, our questionnaire did not have items about exclusive breastfeeding, and thus, we could not separate breastfeeding duration into exclusive and partial durations. Moreover, no previous studies that we have cited in the revised or the previous manuscript have shown that exclusive breastfeeding is more effective than partial breastfeeding in reducing the maternal risks of metabolic syndrome or cardiovascular risk factors. The article suggested by Reviewer #3 examined the association between breastfeeding history and cardiovascular risk factors in children (Int J Obes (Lond). 2019;43(8):1568-1577.), and thus, we have not cited this article in the revised manuscript.

(4) *Line 214: Why is this test not a linear regression? Didn't outcome and exposure have a normal distribution?*

**Response:** Quantile-quantile plots indicated deviation from normality in all the continuous variables. Therefore, we conducted the Kruskal-Wallis test rather than linear regression to examine the associations between the total breastfeeding duration and the continuous variables (lines 219–222).

(5) *Line 226: Even if it was the median age of the study, it does not characterize a strong justification regarding the stratification of the sample. I strongly suggest revising. Maybe it would be better to use more usual maternal age stratifications, separating younger and older mothers.*

**Response:** We have changed the cut-off of the participants’ age from 57 to 55 years old as a usual maternal age in the revised manuscript (lines 235–239). The results of the reanalysis did not materially change from those of the previous manuscript.

(6) *Line 235: This categorization of breastfeeding weeks is too confusing even to refer to the results of the study.*

**Response:** As mentioned in the response to Reviewers #2’s comment (2), we have changed our breastfeeding duration measured in weeks to that measured in months. Additionally, we have again stated the categories of total breastfeeding duration at the beginning of the Results (lines 282–284).

(7) *Table 1. It was not mentioned in the methods that there would be a description of the sample according to the breastfeeding categories. Indicate at the bottom of the table which test is used for p-value.*

**Response:** In the previous and the revised manuscript (lines 220–223), we have written that associations between participant characteristics and the total breastfeeding duration were examined using the Kruskal-Wallis test for continuous variables and the chi-square test of independence for categorical variables. In accordance with this comment, we have added text to indicate that participants’ characteristics were expressed according to the categories of total breastfeeding duration (lines 217–219). We have also added the types of statistical tests to Table 1 in the revised manuscript.

(8) *Table 2. I confess that this variable (Total breastfeeding duration weeks…) is still confused. The authors have enough information, this variable hinders more than helps in the interpretation of results.*

**Response:** Our questionnaire asked about the longest breastfeeding duration for one child among all children and the number of breastfed children, but did not ask about breastfeeding duration for each child. The longest breastfeeding duration ignores breastfeeding durations that mothers could have experienced when they raised other children. Moreover, the number of breastfed children ignores all breastfeeding durations that mothers experienced. To overcome these limitations, we have introduced the variable “total breastfeeding duration.” As pointed out in comment (7) of Reviewer #2, the total breastfeeding duration also has a potential limitation due to misclassification, and thus, we have added this point to the Discussion of the revised manuscript (lines 498–501).

(9) *Line 417: "Finally, the cross-sectional design of our study prevented exclusion of recall bias and reverse causality". Why? Need to justify this claim.*

**Response:** We have added the potential mechanism causing recall bias in the revised manuscript (lines 508–511). The mechanisms of reverse causality are described in the previous and the revised manuscript (lines 501–503). Regarding reverse causality, previous meta-analyses of observational studies showed that mothers with higher body mass index before pregnancy are less likely to initiate or maintain exclusive breastfeeding. In the present study, however, the body mass index at about 20 years old was rather positively associated with all the indices of breastfeeding history (lines 399–402).

(10) *The authors must present a figure with the study sample flowchart, thus facilitating the understanding of the entire sample selection.*

**Response:** We have added Figure 1, which is a study sample flowchart, to the revised manuscript.

(11) *The number of references is extravagant! I suggest reviewing and reducing.*

**Response:** We have reduced the number of references in the revised manuscript from 80 to 53.

(12) *My concern is that they are adjusting for mediator in the wrong way. In addition, you need to check the need for all the variables presented.*

**Response:** We have selected our covariates based on previous studies that reported the associations between these variables and the risk of metabolic syndrome. However, one cohort study reported the association between breastfeeding and early natural menopause (JAMA Network Open. 2020;3(1):e1919615), and a meta-analysis of observational studies and randomized controlled trials showed that menopause can increase the risks of metabolic syndrome and its components (Climacteric. 2017;20(6):583-591.) Thus, we have excluded menopausal status from the covariates as a possible mediator. We thought that the other covariates did not mediate the effect of breastfeeding history on the risk of metabolic syndrome and thus have retained these variables. This is because one of them occurred before delivery (i.e., educational attainment) or because we do not have evidence that breastfeeding history affects the remaining factors long after delivery (i.e., age, residential area, parity, smoking status, alcohol consumption, physical activity, and dietary habit, age at first delivery, and history of hormone replacement therapy).

(13) *Authors need to consider the idea of creating a variable of exclusive breastfeeding and total breastfeeding, there are differences.*

**Response:** Regretfully, as stated in the previous and the revised manuscript (lines 161–163), our questionnaire did not have items concerning exclusive breastfeeding, which has prevented examination of this variable.

(14) *To justify the stratifications performed (maternal age, for example) the authors need to test and present the findings for interaction.*

**Response:** Concerning the associations between breastfeeding history and metabolic syndrome, which was our primary outcome, we have conducted likelihood ratio tests for the product terms of breastfeeding history and age, and all the results were statistically significant (lines 239–242 and 336–340).

Finally, we must report that the numbers of participants classified as missing in several covariates (alcohol consumption, daily total and leisure-time physical activity, and the modified Japanese Diet Index) (Table 1) and those having dyslipidemia (Table 3) have decreased in the revised manuscript. The former occurred because of the incorrect treatment of missing values, and the latter occurred due to a coding error defining dyslipidemia. When we calculate alcohol consumption, daily total and leisure-time physical activity, and the modified Japanese Diet Index, missing values in some items should be treated as zero based on the rules of questionnaires.

Again, we would like to thank the editors and the reviewers for their valuable comments. We hope that the revised manuscript is now suitable for publication in *PLOS ONE*.

Sincerely,

Takashi Matsunaga, MMS, RPT

Department of Preventive Medicine,

Nagoya University Graduate School of Medicine
