# Supplementary material for: Associations of breastfeeding history with metabolic syndrome and cardiovascular risk factors in community-dwelling parous women: The Japan Multi-Institutional Collaborative Cohort Study
Source: PLoS One. 2022 Jan 19;17(1):e0262252. doi: 10.1371/journal.pone.0262252 (PMC8769371; doi:10.1371/journal.pone.0262252)
Supplement: Attachment — Submitted filename: Response To Reviewers.docx [file pone.0262252.s005.docx]

August 28, 2021

Antonio Palazón-Bru, Ph.D.

Academic Editor

*PLOS ONE*

Dear Dr Palazón-Bru:

**Responses to the reviewers’ comments**

To facilitate your review of our revisions, the following are our point-by-point responses to the questions and comments provided in your e-mail dated July 23, 2021. Amendments that we have made are shown in colored text throughout the Revised Article with Track Changes.

Reviewer #2’s comments

Abstract

(1) *Line 66 Cardiovascular risk factors could be listed for added clarity.*

**Response:** We have added lists of the cardiovascular risk factors (Lines 66–67 in the unmarked manuscript).

(2) *Results Suggest adding a sentence about findings in women >55 years.*

**Response:** Following this suggestion, we have added a sentence about the results in women >55 years of age (Lines 80–82).

Methods

(3) *Lines 147-148 Please provide a reference.*

**Response:** We have included a relevant reference of a systematic review (Line 150–152).

(4) *Lines 191-200 How were the categories for alcohol intake and physical activity derived, and for women <55 and ≥55 years? Please provide any relevant references.*

**Response:** According to this recommendation, we have added explanations that these covariates had been categorized based on the distributions of the present population and the cut-offs generally agreed with those reported by previous Japanese studies (Lines 210–212).

Results

(5) *Tables could not be seen in their entirety (perhaps a layout issue) which made review difficult.*

**Response:** In the revised article, we have embedded the tables to fit in one page, but this made the fonts rather small. Thus, we also have submitted the tables as supplementary materials (S Tables 1–3.xlsx). If the reviewers cannot read the fonts of the tables in the main text, please see the supplementary materials.

Discussion

(6) *While the authors have made an effort to make additions to the discussion based on comments received, these additions could be written more succinctly, especially lines 471-484.*

**Response:** We assume that reviewer #2 means the part concerning biological mechanisms behind the inverse associations of breastfeeding with metabolic syndrome and cardiovascular risk factors and have written this part as succinctly as possible (Lines 491–505).

(7) *As previously raised, the total breastfeeding duration variable is limited as it is based on the product of the number of breastfed children and the longest breastfeeding duration in one child, and this variable may not accurately reflect the overall breastfeeding duration across children as breastfeeding duration may vary across children from the same mother. Is there any literature about how breastfeeding duration may vary among children from the same mother, in Japan or other similar countries? Please discuss the limitation of the total breastfeeding duration variable in light of any additional insights provided by relevant literature.*

**Response:** We have included the results of a national survey in Japan and its limitations. Moreover, we have mentioned that the total breastfeeding duration of the present study probably had overestimated the true duration, and that the direction of the bias derived from this overestimation is difficult to ascertain. (Lines 516–521).

Reviewer #3’s comments

(1) *The issues about breastfeeding are fragile, in addition to not having information on exclusive breastfeeding that is strongly associated with cardiometabolic outcomes.*

*Reference: Victora CG, Bahl R, Barros AJ, França GV, Horton S, Krasevec J, Murch S, Sankar MJ, Walker N, Rollins NC; Lancet Breastfeeding Series Group. Breastfeeding in the 21st century: epidemiology, mechanisms, and lifelong effect. Lancet. 2016 Jan 30;387(10017):475-90. doi: 10.1016/S0140-6736(15)01024-7. PMID: 26869575.*

**Response:** The article suggested by reviewer #3 states that a long breastfeeding duration can lead to lower risks of breast and ovarian cancers, osteoporosis, and type 2 diabetes mellitus in mothers, but it does not compare the effects of exclusive breastfeeding to those of partial breastfeeding in reducing the risks of these diseases (“Effects on women who breastfed” part on the table and pages 484–485 in the suggested article). We have cited this article in the Discussion, but have stated that few previous studies examined this issue related to the maternal risks of metabolic syndrome and cardiovascular risk factors (Lines 521–527).

(2) *I suggest that the authors work with the continuous breastfeeding duration variable as well and not in tertiles.*

**Response:** Following this suggestion, we conducted a sensitivity analysis that examined the associations between each measurement of breastfeeding history and metabolic syndrome by incorporating these measurements into models as continuous variables. The results remained almost the same as those of the trend tests that incorporated ordinal variables measuring breastfeeding duration using tertiles (Lines 250–254, 392–395, and S1 Table). We have also mentioned that the measurements of breastfeeding history were categorized to deal with potential nonlinear associations in the Exposure part of the Methods (Lines 166–170).

(3) *Tables are cut, it was not possible to perform a full evaluation.*

**Response:** In the revised article, we have embedded the tables to fit in one page, but this made the fonts rather small. Thus, we also have submitted the tables as supplementary materials (S Tables 1–3.xlsx). If the reviewers cannot read the fonts of the tables in the main text, please see the supplementary materials.

(4) *Review analytics to avoid adjustments for association mediators.*

**Response:** We agree that several confounding factors in our study (smoking status, alcohol consumption, daily total physical activity, leisure-time physical activity, the modified Japanese Diet Index, and history of hormone replacement therapy) probably followed breastfeeding history in time and could be association mediators. Thus, we conducted a sensitivity analysis that examined associations between each indicator of breastfeeding history and metabolic syndrome by incorporating only covariates that had preceded breastfeeding history in time (Lines 254–257). The results were generally unchanged from those in the multivariable-adjusted model of Table 2 (Lines 395–399 and S2 Table). We also have added a discussion about a bias developed from adjusting for potential mediators (Lines 540–548). Finally, in the Discussion, we have mentioned the need for prospective assessment of potential confounding factors to obviate this bias (Lines 549–551).

(5) *Regarding the temporality of events, one should consider the possibility that some of the evaluated outcomes precede the breastfeeding behavior.*

**Response:** In response to this comment, we added a discussion about another possible example of reverse causality other than that due to obesity before pregnancy, where gestational diabetes may prevent the initiation and maintenance of breastfeeding (Lines 531–534).

(6) *Study limitations should be described and discussed only in the discussion section.*

**Response:** We assume that reviewer #3 is referring to the part about no data on exclusive breastfeeding and the approximation of the total breastfeeding duration stated in the Methods. We have added this limitation in the Discussion (Lines 512–527). For clarity, however, we also retained the description in the Exposure part of the Methods (Lines 162–166).

Again, we would like to thank the editors and the reviewers for their valuable comments. We hope that the revised manuscript is now suitable for publication in *PLOS ONE*.

Sincerely,

Takashi Matsunaga, MMS, RPT

Department of Preventive Medicine,

Nagoya University Graduate School of Medicine
