# Supplementary material for: Associations of breastfeeding history with metabolic syndrome and cardiovascular risk factors in community-dwelling parous women: The Japan Multi-Institutional Collaborative Cohort Study
Source: PLoS One. 2022 Jan 19;17(1):e0262252. doi: 10.1371/journal.pone.0262252 (PMC8769371; doi:10.1371/journal.pone.0262252)
Supplement: Attachment — Submitted filename: Response To Reviewers.docx [file pone.0262252.s006.docx]

October 29, 2021

Antonio Palazón-Bru, Ph.D.

Academic Editor

*PLOS ONE*

Dear Dr Palazón-Bru:

Thank you for your e-mail dated September 30, 2021. We appreciated the reviewers’ thoughtful comments. To facilitate your review of our revisions, the following are our point-by-point responses to the questions and comments provided in your e-mail. Amendments that we have made are shown in colored text throughout the “Revised Article with Track Changes. docx.” file.

**Responses to the reviewers’ comments**

Reviewer #2’s comments

*Discussion*

*Line 443 The sentence states that "although no systematic review or meta-analysis has been conducted". However, I am aware of a systematic review that examines breastfeeding and maternal cardiovascular risk factors and cardiovascular disease, and that mentions effects based on age. Breastfeeding and maternal cardiovascular risk factors and outcomes: A systematic review. PLoS One. 2017; 12(11): e0187923. The authors could check for other systematic reviews/meta-analyses, just in case.*

**Response:** We apologize for having overlooked the suggested article and cited this article in the Discussion in the revised manuscript (ref. 37, lines 446–449). Another systematic review investigated whether the duration between delivery and the study or length of follow-up influences the association between breastfeeding and hypertension with a cut-off of 6 months (Bonifacino E et al. Breastfeed Med. 2018;13(9):578-588.) However, this cut-off is too short to examine the effects of breastfeeding on metabolic syndrome in middle-aged or older women, and thus we did not cite this systematic review.

Reviewer #3’s comments

If the tables cannot be seen in the main text, please refer to the Excel files uploaded to the submission and review system.

*(2) I suggest that the authors work with the continuous breastfeeding duration variable as well and*

*not in tertiles. It would be interesting to present these sensitivity analyses.*

**Response:** In the previous and the revised manuscript, we have conducted the suggested sensitivity analysis (S1 Table). In both the younger and the older age groups, the results remained almost the same as those of the trend tests in the main analysis (Table 2). The number of breastfed children was inversely associated with metabolic syndrome in the younger age group, whereas the longest and total breastfeeding duration was positively correlated with the syndrome in the older age group. In all participants, the longest and total breastfeeding duration was also correlated with the syndrome in this sensitivity analysis, which was different from results of the main analysis with the tertiles (S1 Table and lines 250–254 and 392–400).

*(4) Review analytics to* *avoid adjustments for association mediators. It would be interesting to present these sensitivity analyses. Adjustment for mediators is considered a serious error because such variables are in the middle of the causal chain between exposure and outcome. Considering this, if the authors decide to keep these adjustments, my suggestion is that a robust mediation analysis be performed.*

**Response:** As a sensitivity analysis, we excluded potential mediators from the covariates (S2 Table). The directions of associations were unchanged from those of the main analysis as we presented the result in the previous and the present manuscript (Table 2).

Regarding mediation analysis, however, we disagree with reviewer #3’s suggestion for three reasons. First, adjustments for descendants of exposure induce biases, but those for factors in the future of exposure could reduce biases (Hernán MA, Robins JM. Causal inference: what if. pp95. Available from: https://cdn1.sph.harvard.edu/wp-content/uploads/sites/1268/2021/03/

ciwhatif_hernanrobins_30mar21.pdf). As a simulation study has shown, if the covariates of the present study are affected by unmeasured confounding variables and the effects of confounding are strong, not adjusting for these covariates may lead to residual confounding even if these covariates are also mediators. This simulation study recommends presenting both results adjusted and unadjusted for potential mediators that are also descendants of confounding variables when only variables that concede exposure were measured and sufficient knowledge is not available about relationships between variables examined in studies (Groenwold RHH et al. Epidemiology. 2021;32(2):194-201.) Following this recommendation, we have retained all the covariates in main models and conducted the sensitivity analysis mentioned above (lines 554–562). The directions of the results were unchanged when we excluded potential mediators from the covariates (S2 Table and lines 400–404). Second, the present study aimed to investigate neither the direct nor indirect effects but the total effect of breastfeeding on metabolic syndrome. The investigation of total effects does not require mediation analysis. Even if the covariates of the present study are potent mediators, the additional analysis provides total effect estimates that avoid the bias from the adjustments for these covariates (S2 Table). Third, our study measured few potential confounding factors that preceded breastfeeding history. Even if we conduct mediation analysis, we should not obtain valid estimates of the mediating effects without sufficient adjustments for confounding factors. Thus, we did not conduct a mediation analysis.

Again, we would like to thank the editors and the reviewers for their valuable comments. We hope that the revised manuscript is now suitable for publication in *PLOS ONE*.

Sincerely,

Takashi Matsunaga, MMS, RPT

Department of Preventive Medicine,

Nagoya University Graduate School of Medicine
